# Nitrogen Management in a Maize-Groundnut Crop Rotation of Humid Tropics: Effect on N2O Emission

**DOI:** 10.1100/tsw.2001.453

**Published:** 2001-12-12

**Authors:** M.I. Khalil, A.B. Rosenani, O. Van Cleemput, C.I. Fauziah, J. Shamshuddin

**Affiliations:** Soil Science Division, Bangladesh Institute of Nuclear Agriculture, Mymensingh, Bangladesh

**Keywords:** N sources, nitrification, humid tropics, maizegroundnut crop rotation

## Abstract

Development of appropriate land management techniques to attain sustainability and increase the N use efficiency of crops in the tropics has been gaining momentum. The nitrous oxides (N2Os) affect global climate change and its contribution from N and C management systems is of great significance. Thus, N transformations and N2O emission during maize-groundnut crop rotation managed with various N sources were studied. Accumulation of nitrate (NO3 –) and its disappearance happened immediately after addition of various N sources, showing liming effect. The mineral N retained for 2–4 weeks depending on the type and amount of N application. The chicken manure showed rapid nitrification in the first week after application during the fallow period, leading to a maximum N2O flux of 9889 μg N2O-N m–2 day– 1. The same plots showed a residual effect by emitting the highest N2O (4053 μg N2O-N m–2 day– 1) during maize cultivation supplied with a halfrate of N fertilizer. Application of N fertilizer only or in combination with crop residues exhibited either lowered fluxes or caused a sink during the groundnut and fallow periods due to small availability of substrates and/or low water-filled pore space (<40%). The annual N2O emission ranged from 1.41 to 3.94 kg N2O-N ha–1; the highest was estimated from the chicken manure plus crop residues and half-rate of inorganic N-amended plots. Results indicates a greater influence of chicken manure on the N transformations and thereby N2O emission.

## INTRODUCTION

At present, gaseous emissions of reactive nitrogen (N) are a worldwide concern and are increasing, particularly nitrous oxide ($N_2O$) that causes global warming and ozone layer depletion[1]. Increasing the productivity of agriculture, particularly from tropical soils, will require increased use of N amendments. Soil-applied N will serve as a feedstock for soil N reactions, including those that emit gaseous N. The N cycle in agricultural systems is influenced by many factors, namely N supplying capacity of soils, which depends mostly on the indigenous soil organic matter, addition of organic residue, and the various soil environmental factors: moisture, temperature, aeration, and pH. The $N_2O$ production and diffusion is considerable upon irrigation/rainfall events under upland conditions by changing the soil physical-chemical properties or by affecting soil-gas diffusivity and microbial activity and subsequent N gas production and efflux[2,3].

Most applied studies of agricultural N cycles, budgets, and estimates of gaseous emissions have been reported from research conducted in temperate areas or from flooded-rice culture in tropical areas. The humid tropics cover large areas and necessitate higher N inputs to improve agricultural productivity. There is a dearth of information from tropical upland agricultural systems, particularly from crop residue and animal manure applied to agricultural soils[4]. Moreover, the weather of humid tropics is considered to be conducive to influence gaseous and leaching losses of N with poor N use efficiency by the crops. Therefore, this study was carried out to estimate the emission of $N_2O$ from an upland cropping system applied with both inorganic and organic N (as crop residue/amendment) fertilizers.

## MATERIALS AND METHODS

A field experiment was conducted at the Universiti Putra Malaysia experimental farm (101° 42' E, 3°02' N). The well-drained soil belongs to the Bungor Series (loamy, kaolinitic, isohyperthermic family of Typic Paleudults). The $pH_{H_2O}$ is low (5.3) and the N, organic C content, and CEC are 0.13%, 1.25%, and 6.86 $cmol_c$ $kg^{-1}$, respectively. The daily pattern of rainfall and air temperature during the study period is shown in Fig. 1. The average air humidity during the study period was about 90%, ranging from 72 to 100%. The rainfall was more or less well distributed throughout the year. Total rainfall during the investigation period (365 days, 1998–99) was 2293 m. The minimum and maximum air temperature was 19.5 and 34.5°C with a yearly average of 20.7 and 31.9°C, respectively.

The field study was carried out from November 1998 to November 1999 in order to cover a 1-year period. It started with the groundnut (day 0–90), followed by a fallow period (day 90–192), the maize growing period (day 192–285), and again followed by a fallow period (day 285–365). During the last 2 years, it was supplied with both inorganic fertilizer ($[NH_4]_2SO_4$) and crop residues/chicken manure. The recommended N dose was 30 kg N $ha^{-1}$ for groundnut and 150 kg N $ha^{-1}$ for maize. All experimental units received P and K at the same rate of 90 kg $ha^{-1}$ as triple-super phosphate and muriate of potash, respectively during both the groundnut and maize period. Crop residues (8–10 t $ha^{-1}$ on average) of maize (%C = 48.5, %N = 1.57, and C/N = 34.4) and groundnut (%C = 46.0, %N = 2.68, and C/N = 19.3) were spread after the harvest of each crop. Chicken manure with a moisture content of 22.5% (%C = 20.8, %N = 2.16, and C/N = 9.6) at a rate of 10 t $ha^{-1}$ (on wet basis, 168 kg N $ha^{-1}$) was applied before maize cultivation only. The field was limed with ground magnesium limestone at a rate of 2 t $ha^{-1}$ before each crop cycle. The treatments were as follows: $T_1$: recommended inorganic N + crop residues (NC), $T_2$: recommended inorganic N only (RN), and $T_3$: half-dose of recommended inorganic N + crop residues + chicken manure (NCM). The experiment was conducted in a randomized complete block (RCB) design with four replications for each treatment. The size of each plot was 20 × 8 m with a total area of 3000 $m^2$. The chemical fertilizers were applied in furrows for both the crops. For maize, $(NH_4)_2SO_4$ was applied in two splits (two thirds at sowing in furrows and one third at silking stage by single-band placement) as per treatments. The maize (4.6 t $ha^{-1}$ on dry basis or 72 kg N $ha^{-1}$) and groundnut (3.0 t $ha^{-1}$ on dry basis or 70 kg N $ha^{-1}$) residues were spread on the field after harvest of each crop and incorporated 1 week before the cultivation of the succeeding crop. *Rhizobium* inoculated groundnut (*Arachis hypogae*) seeds were sown in furrows after application of the fertilizers on

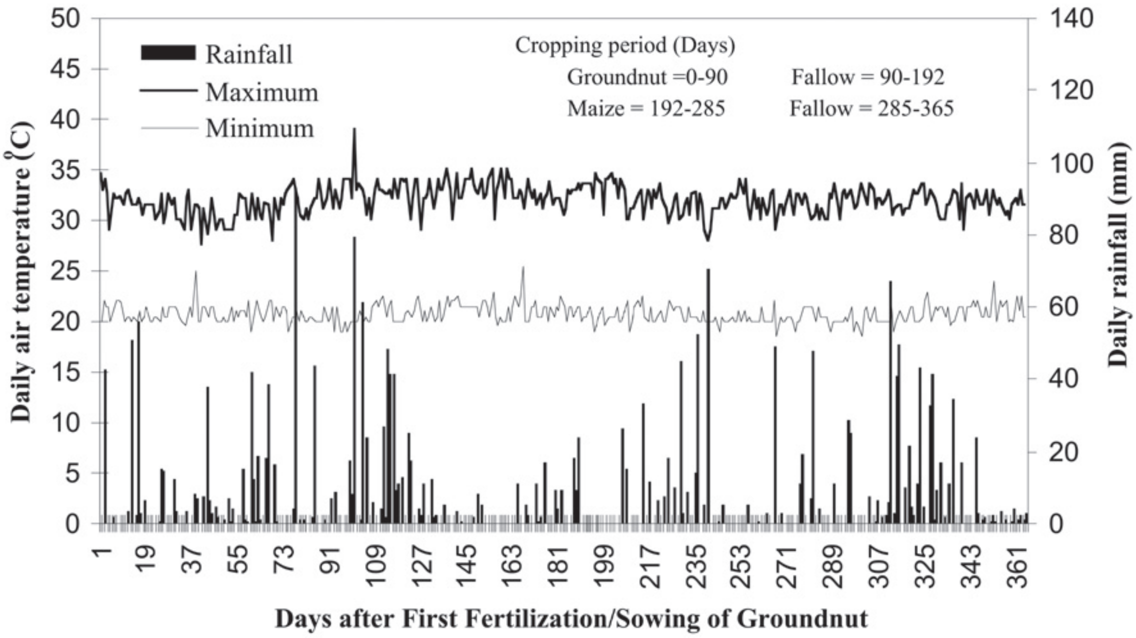

**FIGURE 1.** Daily minimum and maximum air temperature and rainfall during the groundnut-fallow-maize-fallow period (day 1 corresponds to November 19, 1998).

November 18, 1998, and harvested on February 19, 1999. Maize (*Zea mays*) seeds were sown on May 29, 1999, and harvested on September 1, 1999.

Gas samples were collected using a closed box (cross-section: 184 cm$^2$, height: 8 cm) that fitted with a vented perspex lid consisting of a rubber septum at the middle. The gas chambers were placed in between the plants. Gas samples were taken in the morning (9–11 h) through the use of a double-sided needle in 10-ml Vacutainers® (Beckton Dickinson, U.S.) at 0, 15, and 30 min after closing the chamber. After sampling, the chambers were kept open until the next gas collection. Collection of gas samples started the day following fertilizer application/sowing of groundnut seeds. The samples were analysed by gas chromatography (Model HP 6890) using $^{63}$Ni electron capture detector and calculated $N_2O$ fluxes[5].

Composite soil samples were collected at each day of gas sampling to a depth of 15 cm to analyze for $NH_4^+$-N, $NO_3^-$-N, and $NO_2^-$-N. Soil pH and water-soluble organic carbon (WSOC)[6] at a soil:water ratio of 1:2.5 was determined as well. Soil-water content was measured gravimetrically at each gas sampling time, and the corresponding water-filled pore spaces (WFPS) were calculated. Statistical analysis was performed using the statistical package SAS (1989). Annual $N_2O$ emission was calculated by integrating a specific area under the $N_2O$ flux curve during the crops growth/fallow periods during a 1-year study.

## RESULTS AND DISCUSSION

### $N_2O$ Fluxes during Groundnut Growth Period (Day 1–90)

The $N_2O$ fluxes varied with treatments during the 1-year study on a maize-groundnut cropping pattern and the succeeding fallow period after each cropping (Fig. 2). The gas sampling for the $N_2O$ measurement was started 1 day after sowing and fertilization of groundnut on November 18, 1998. The groundnut crop was harvested at day 90 after sowing. The highest peaks of $N_2O$ flux, ranging from 770 to 983 µg $N_2O$-N m$^{-2}$ day$^{-1}$, were detected within 2 weeks of N fertilizer application during the groundnut growth period. The highest flux was found in the NC treatment receiving N fertilizer and crop residues previously. The high $N_2O$ fluxes immediately after application of the N fertilizer probably corresponded to the decrease in $NH_4^+$ concentration and the resulting $NO_3^-$ accumulation during the initial periods (Figs. 3a and b). It was followed by $NO_3^-$ disappearance, i.e., denitrification. However, the trend of N transformations was mostly similar irrespective of treatments. It may be attributed to the influence of liming applied before cultivation of the groundnut. The $NO_2^-$ accumulation was very low (data not shown). The WFPS was more than 50%, which might be conducive to both nitrification and denitrification during the groundnut growing period (Fig. 3c). The amount of mineral N decreased considerably, 2 weeks after fertilization, producing smaller $N_2O$ peaks, due to the small availability of mineral N and the resultant small nitrification/denitrification. It was observed from a soybean or alfalfa plot that low N (20 or 40 kg N ha$^{-1}$) increased the emission to a small extent and did not vary significantly with the control plots (having residual N only)[7].

### $N_2O$ Fluxes During Fallow Period After Groundnut (Day 90–192)

During fallow period after the groundnut harvest, the highest fluxes were detected at day 120 from NCM (652 µg $N_2O$-N m$^{-2}$ day$^{-1}$), followed by NC (614 µg $N_2O$-N m$^{-2}$ day$^{-1}$) (Fig. 2). Both of them continued to emit at higher rates up to 2 weeks after crop

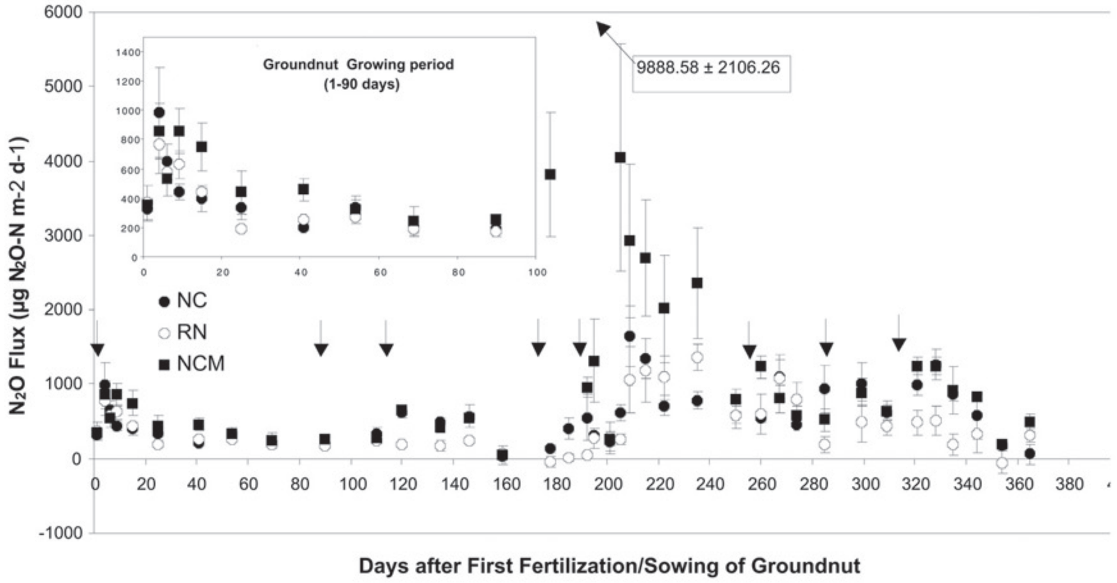

**FIGURE 2.** $N_2O$ fluxes during the entire maize-groundnut rotation as influenced by inorganic and organic N sources, day 1 corresponds to November 18, 1998 (NC = recommended N + crop residue, RN = recommended N only, and NCM = recommended N + crop residue + chicken manure). Arrows from left indicate time of groundnut sowing and fertilizer application, harvest of groundnut, spread of groundnut residue, chicken manure application, sowing of maize and 2/3 N + other fertilizers application, application of 1/3 N, harvest of maize and spread of maize residue, respectively. Vertical bars indicate standard error.

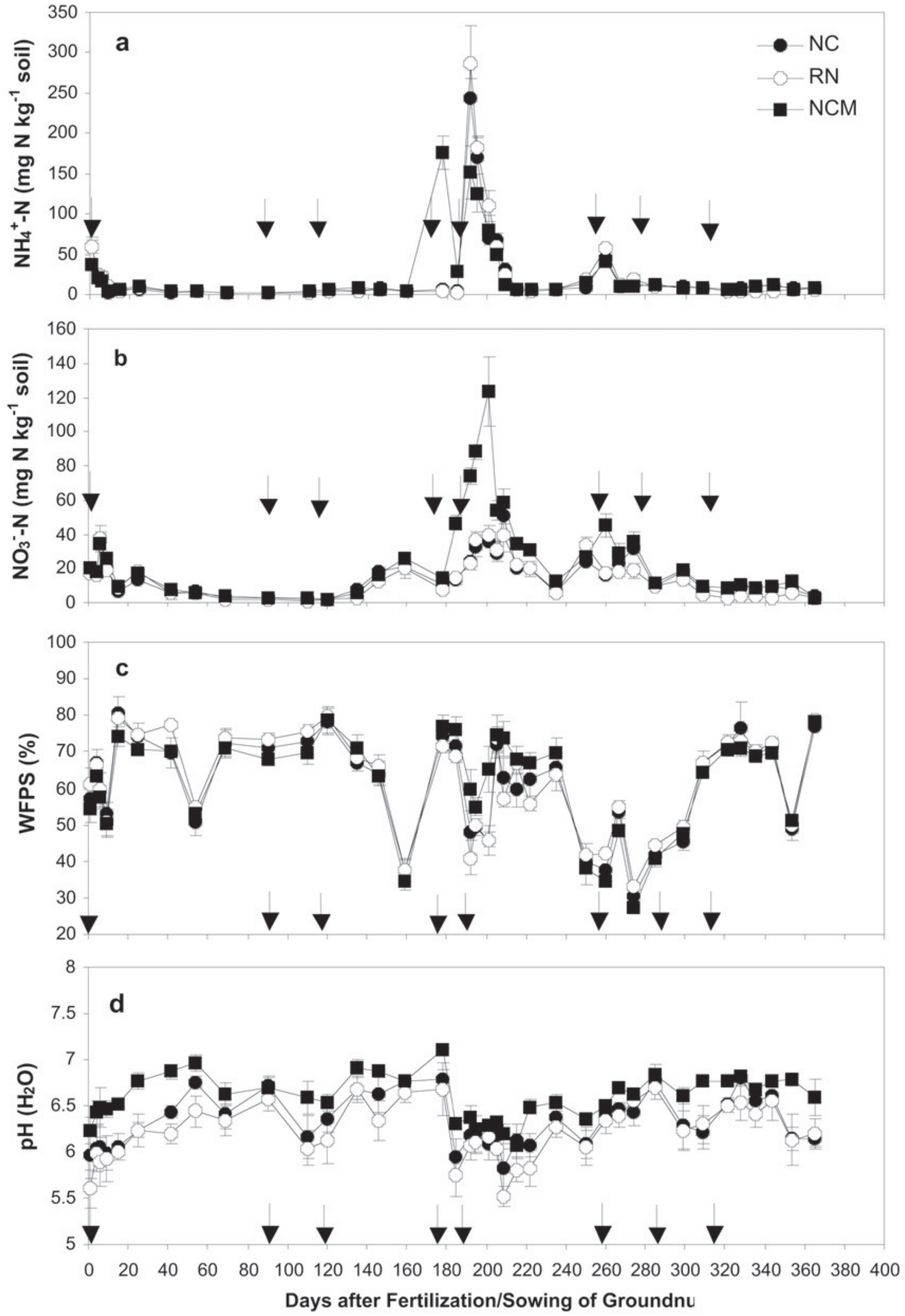

**FIGURE 3**. Changes in (a) $NH_4^+$-N, (b) $NO_3^-$-N, (c) WFPS NCM = half of recommended, and (d) soil pH with time during the groundnut-fallow-maize-fallow period as influenced by inorganic and organic N fertilizers (NC = recommended N + crop residue, RN = recommended N only and NCM = recommended N + crop residue + chicken manure). Arrows indicate the day of groundnut sowing/fertilization, groundnut harvest, spread of groundnut residue, chicken manure application, maize sowing/fertilization (2/3 N), 1/3 N application, maize harvest and maize residue application. Vertical bars indicate standard error.

residue application. Application of chicken manure showed a greater increase of $N_2O$ emission immediately at the measurement day of 178 and 185. The fluxes ranged from 3820 to 9889 µg $N_2O$-N $m^{-2}$ $day^{-1}$ and varied significantly ($p \leq 0.01$). At the same measurement period, the fallow plot RN showed either the lowest (10 µg $N_2O$-N $m^{-2}$ $day^{-1}$) emission or caused a sink (47 µg $N_2O$-N $m^{-2}$ $day^{-1}$). The very high $N_2O$ flux in the treatment amended with chicken manure may coincide with the rapid consumption or oxidation of high amounts of $NH_4^+$-N by its oxidizers in the chicken manure and the resultant large nitrification (Figs. 3a and b). The poultry manure, with a low C:N ratio, increases the respiratory activity and mineralization as compared with other litter types[8]. The higher availability of $NH_4^+$-N was not a limiting factor for the initial increase of nitrification and coupled nitrification-denitrification[9] and thereby $N_2O$ emission. With a high accumulation of $NO_3^-$ and $NO_2^-$ during both nitrification and marginal denitrification, $N_2O$ will be the dominant gas to release[10,11]. Availability of WSOC and favourable pH (Fig. 3d) under ascending WFPS (~76%) during the same period could also stimulate the nitrification and denitrification processes, to some extent, at the later periods (Fig. 3c). The highest peak was detected while the WSOC (data not shown) and pH decreased significantly during the nitrification process. Indeed, soil pH exerted a complex control on microbial activity related to nitrification and denitrification. An increased concentration of $NO_3^-$, and the acidity produced by nitrification, favours the production of $N_2O$ relative to $N_2$[12,13,14]. A similar, but lower, peak for $N_2O$ emission (7900 μg $N_2O$-N $m^{-2}$ $day^{-1}$) from a manure-amended soil has also been reported[15].

## $N_2O$ Fluxes During Maize Growth Period (Day 192–285)

Maize seeds were sown at day 192, supplied with 100 kg N $ha^{-1}$ for NC and RN and one half of the amount for NCM. At day 192, a significant ($p < 0.05$) difference of $N_2O$ flux was found among the treatments (Fig. 2). An increasing trend of $N_2O$ flux was detected after 2 weeks of N fertilizer application. Application of the remaining 50 kg N $ha^{-1}$ of the N fertilizer increased the $N_2O$ emission insignificantly. Application of a high amount (100 kg N $ha^{-1}$) of $N_4$+ fertilizer during maize sowing did not show an immediate high release of $N_2O$ because of either a low $NO_3^-$ content or low WFPS (<50%). Until day 201, nitrification was probably the dominant process for $N_2O$ emission due to the low WFPS (~50%) and thereby a sharp release of $N_2O$ was detected due to the increased amount of $NO_3^-$ disappearance, i.e., denitrification (Figs. 3b and c). This was favoured by a higher moisture content (WFPS > 70%). Accordingly, the highest peak for $N_2O$ flux was observed at day 205, mainly from the NCM treatment (4043 μg $N_2O$-N $m^{-2}$ $day^{-1}$), since a maximum $N_2O$ emission from agricultural soils was generally observed within 2–3 weeks after fertilizer application[16]. But it was lower than the highest peak detected immediately after addition of chicken manure during the fallow period after groundnut. This indicates the contribution of chicken manure applied during the fallow period, influencing nitrification through consumption of $NH_4^+$ and nitrification-denitrification processes, though it fluctuated with time (Figs. 3a and b). Higher $N_2O$ fluxes have also been reported elsewhere under maize cover due to application of pig slurry[17].

The $N_2O$ emissions were mostly through probable denitrification along with some nitrification-induced emission, and the high rates of emission coincided with the periods when the $NO_3^-$-N and soil water content were relatively high. In comparison with the other treatments (NC and RN), the combination of inorganic N fertilizer and organic residues along with the chicken manure contributed to more $N_2O$ emission[18]. The next highest peak (1652 μg $N_2O$-N $m^{-2}$ $day^{-1}$) was detected at day 209 in NC, which received crop residue after the harvest of the previous groundnut crop, but it was lower than in the NCM treatment. This indicates that there was an influence of crop residue also on $N_2O$ emission. Though crop residues stimulated $NO_3^-$ disappearance to release a higher $N_2O$ amount than nitrification (Fig. 3b), it was favoured by a high WFPS (more than 60%) and pH ~ 6.0 (Figs. 3c and d). Many researchers agreed with these findings. Application of organic residues, particularly easily decomposable ones, enhanced $NO_3^-$ reduction under aerobic conditions and through anaerobic microsites, thereby influencing $N_2O$ emission[5,9,19,20,21].

## $N_2O$ Fluxes During Fallow Period After Maize (Day 285–365)

During fallow period after the maize, the highest peak was detected in NC (1009 μg $N_2O$-N $m^{-2}$ $day^{-1}$) followed by NCM (892 μg $N_2O$-N $m^{-2}$ $day^{-1}$) during the initial fallow period while the maize plants remained standing (Fig. 2). There was an immediate and significant response of maize residue application on the $N_2O$ flux at day 321 and 328. The NC treatment showed the highest peak (1265 μg $N_2O$-N $m^{-2}$ $day^{-1}$) in association with NCM (1247 μg $N_2O$-N $m^{-2}$ $day^{-1}$) at day 328, though the latter emitted the same amount of $N_2O$ at day 321. At the end of the fallow period after maize, the release of $N_2O$ was very low in NC and NCM (180 to 188 μg $N_2O$-N $m^{-2}$ $day^{-1}$), and an $N_2O$ consumption was detected in RN (58 μg $N_2O$-N $m^{-2}$ $day^{-1}$). The fallow period after maize showed higher $N_2O$ fluxes than the fallow period followed by groundnut except immediately after application of chicken manure. This may be attributed to the contribution of the comparatively higher amount of residual N remaining during the fallow period that was applied to the maize field (Figs. 3a and b). The pH (Fig. 3d), availability of WSOC (data not shown) and WFPS of more than 40% (Fig. 3c) during the same period could also influence the nitrification and denitrification processes to some extent. The presence of high residual N during the fallow period after maize might influence the C and N mineralization[22]. It was lower than the peaks formed due to high rates of N fertilizer applied to maize plots. Similar findings have been reported elsewhere[23]. This confirms the importance of the available substrates to produce and release $N_2O$ under favourable environmental conditions.

The plots receiving N fertilizer only during the cropping periods showed a comparatively lower $N_2O$ flux during the fallow periods due to the small availability of substrates or low WFPS (<40%) than the plots amended with crop residues alone or in combination with chicken manure. Low substrate availability and/or rainfall immediately after a dry period also caused sinks at the end of both fallow periods without amendments. It has been reported that the potential for gaseous N losses at WFPS < 40% was low or negative[17,24,25]. These indicate that the soil was able to immobilize atmospheric $N_2O$, but with a very weak efficiency[26]. However, addition of crop residue either alone or with chicken manure influenced the nitrification and denitrification processes.

## Annual $N_2O$ Emission

In Malaysia, temperature fluctuations are minimal throughout the year. Agricultural practices and moisture regime could be the major factors affecting the variations of $N_2O$ fluxes. Therefore, the total $N_2O$ emission was estimated over the total crop growth and fallow periods, with or without amendment of crop residues/chicken manure. The total $N_2O$ emission was significantly higher during the maize growth (93 days) period (1.82 ± 0.20 kg $N_2O$-N $ha^{-1}$) from the NCM treatment that received half of the recommended amount of N fertilizer along with chicken manure and crop residues (Fig. 4). It was followed by the fallow period (102 days) after the groundnut harvest (1.12 ± 0.03 kg $N_2O$-N $ha^{-1}$), which received both groundnut residue and chicken manure. The magnitude of $N_2O$ emissions during both crop growth and fallow

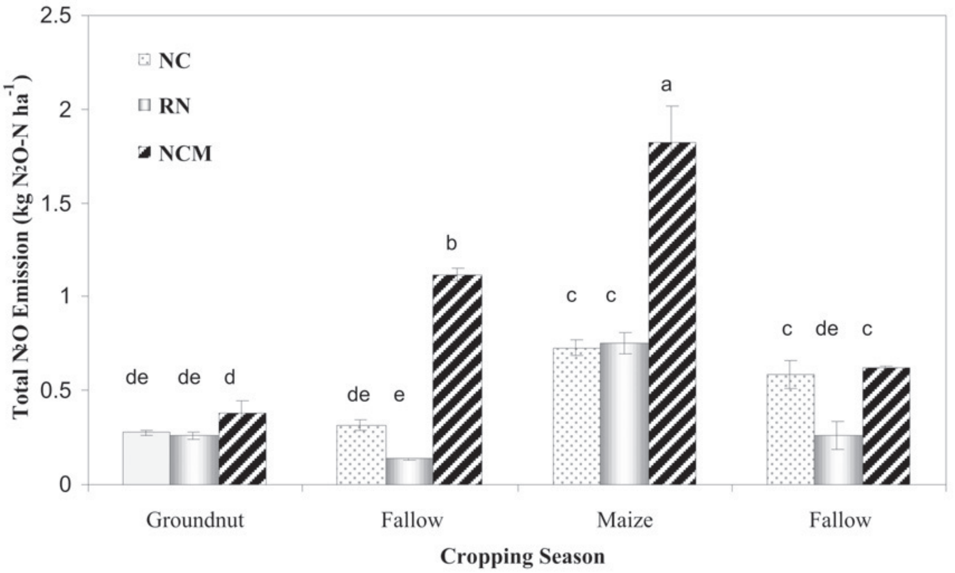

**FIGURE 4**. Total $N_2O$ fluxes as influenced by inorganic and organic N sources during the different growing periods (NC = recommended N + crop residue, RN = recommended N only and NCM = half of recommended N + crop residue + chicken manure). Vertical (thin) bars indicate standard error. Thick bar (s) having common letter (s) do not differ significantly at 5% level of Duncan's Multiple Range Test.

periods was mostly influenced by the availability of mineral N under favourable WFPS[27]. Similarly, the regulatory effects of rainfall and fertilizer application on $N_2O$ emission indicated the important contribution of WFPS and $NO_3^-$ concentrations[24,25]. The trend of $N_2O$ emission under maize cover was in line with the reports of several researchers[5,15,17].

The observed annual $N_2O$ fluxes varied significantly ($p < 0.0001$) during the total maize-groundnut crop rotation period. Table 1 shows that NCM emitted 3.94 ± 0.23 kg $N_2O$-N $ha^{-1}$ $year^{-1}$ followed by NC (1.90 ± 0.08 kg $N_2O$-N $ha^{-1}$ $year^{-1}$). The lowest emission was from the RN treatment supplied with N fertilizer only (1.41 ± 0.07 kg $N_2O$-N $ha^{-1}$ $year^{-1}$). Addition of N in the form of manure or crop residue had more effect on $N_2O$ emissions than fertilizer N addition[28,29]. The increase of total $N_2O$ emission from the NCM and NC treatments over the RN treatment was 180 and 35%, respectively. The relative $N_2O$-N loss of the added N, considering background emission negligible, varied significantly ($p < 0.01$), with the highest loss of 0.99% from the NCM treatment receiving chicken manure, crop residue, and half of N fertilizer. The $N_2O$-N loss was 0.70% from the RN treatment, followed by the NC treatment (0.59%). This also confirms the influence of chicken manure on $N_2O$ emission rather than the addition of crop residue, where the latter showed a lower emission than the plots supplied with N fertilizer only. A similar loss of $N_2O$ from different upland cropping systems has also been observed[30,31]. The yearly estimations of fertilizer-induced $N_2O$ emission were of the same order of the 1.25% $N_2O$-N loss from fertilizers with an uncertainty range of 0.25 to 2.25%[34]. Results indicate that the addition of chicken manure alone or in combination with other N sources could be an important potential source of $N_2O$.

**TABLE 1**
**Annual $N_2O$ Emission and Apparent $N_2O$-N Loss from the Added N Sources During Maize-Groundnut Crop Rotation**

| Treatments | Annual $N_2O$ Emission (kg $N_2O$-N $ha^{-1}$) | $N_2O$-N Loss of the Added N (%) |
|---|---|---|
| NC (Half of recommended N fertilizer + crop residues) | 1.90 ± 0.08 | 0.59 ± 0.03 |
| RN (Half of recommended N fertilizer) | 1.41 ± 0.07 | 0.70 ± 0.09 |
| NCM (Half of recommended N fertilizer + crop residues + chicken manure) | 3.94 ± 0.23 | 0.99 ± 0.05 |

## CONCLUSIONS

The $N_2O$ fluxes from the maize-groundnut rotation varied with the forms and amount of fertilizer N applied, appearing proportional to substrate, i.e., $NO_3^-$ in soil. Addition of the chicken manure and crop residues could influence N transformation processes for a long time and thereby influence $N_2O$ release. However, the retention of mineral N in this soil could be 2–4 weeks after application of N in either sources because of rapid decomposition, high rainfall, and temperature, causing runoff and leaching losses of the applied N. As such the annual $N_2O$ emissions were very small relative to N inputs, and apparently 99% or more of the applied N remained in the crop-soil-water system indicating insignificant importance of $N_2O$ emissions from an agronomic standpoint. The immediate large release of $N_2O$ due to chicken manure suggests to modify the existing time and method of application for better N retention in soil and subsequent N use by crops.

## ACKNOWLEDGEMENTS

This research work was funded jointly by Ghent University, Belgium (VL.I.R Programme) and Universiti Putra Malaysia under a UPM-Ghent University Ph.D Twinning Programme. The authors wish to thank Professor G. Stoops, Ghent University, the coordinator of the programme, and the Belgian and Malaysian governments for financial assistance.

| | | |
|---|---|---|
| Received: | August | 17, 2001 |
| Revised: | November | 15, 2001 |
| Accepted: | November | 15, 2001 |
| Published: | December | 12, 2001 |

## BIOSKETCH

**M. Ibrahim Khalil** is a Senior Scientific Officer with the Bangladesh Institute of Nuclear Agriculture. He holds an M.Sc. Ag. in Soil Science (Bangladesh Agricultural University), a Master in Soil Science and Eremology (Ghent University, Belgium), and a Ph.D. in Soil Chemistry (Universiti Putra Malaysia). His research interests are 1) To measure the emission of greenhouse gases from major cropping systems of the sub-tropics, to identify the causes and determining factors influencing the emission, to make a national inventory and simulation of related models, and to find out better management options to reduce the emission; and 2) to study the dynamics of organic amendments (locally available sources) on the organic matter build-up and sustained crop productivity with emphasis on C discrimination and N synchronization using doubly labeled 13C-15N isotopes.
